# NIR light guided enhanced photoluminescence and temperature sensing in Ho^3+^/Yb^3+^/Bi^3+^ co-doped ZnGa_2_O_4_ phosphor

**DOI:** 10.1038/s41598-021-83644-9

**Published:** 2021-02-18

**Authors:** Monika, Ram Sagar Yadav, Anita Rai, Shyam Bahadur Rai

**Affiliations:** 1 Laser and Spectroscopy Laboratory, Department of Physics, Institute of Science, Banaras Hindu University, Varanasi, 221005 India; 2 Department of Zoology, Institute of Science, Banaras Hindu University, Varanasi, 221005 India; 3 Department of Chemistry, PPN College, Kanpur, 208001 India

**Keywords:** Lasers, LEDs and light sources, Optical materials and structures, Optical physics, Optical techniques, Other photonics

## Abstract

The conversion of NIR light into visible light has been studied in Ho^3+^/Yb^3+^/Bi^3+^ co-doped ZnGa_2_O_4_ phosphor for the first time. The crystallinity and particles size of the phosphor increase through Bi^3+^ doping. The absorption characteristics of Ho^3+^, Yb^3+^ and Bi^3+^ ions are identified by the UV–vis-NIR measurements. The Ho^3+^ doped phosphor produces intense green upconversion (UC) emission under 980 nm excitations. The emission intensity ~ excitation power density plots show contribution of two photons for the UC emissions. The UC intensity of green emission is weak in the Ho^3+^ doped phosphor, which enhances upto 128 and 228 times through co-doping of Yb^3+^ and Yb^3+^/Bi^3+^ ions, respectively. The relative and absolute temperature sensing sensitivities of Ho^3+^/Yb^3+^/5Bi^3+^ co-doped ZnGa_2_O_4_ phosphor are calculated to be 13.6 × 10^−4^ and 14.3 × 10^−4^ K^−1^, respectively. The variation in concentration of Bi^3+^ ion and power density produces excellent color tunability from green to red via yellow regions. The CCT also varies with concentration of Bi^3+^ ion and power density from cool to warm light. The color purity of phosphor is achieved to 98.6% through Bi^3+^ doping. Therefore, the Ho^3+^/Yb^3+^/Bi^3+^:ZnGa_2_O_4_ phosphors can be suitable for UC-based color tunable devices, green light emitting diodes and temperature sensing.

## Introduction

The zinc gallate (ZnGa_2_O_4_) based phosphors are very promising photoluminescent materials due to their unique low phonon energy. This permits large photoluminescence intensity of the lanthanide ions for various exciting applications, such as display devices, field emission display devices (FEDs), temperature sensing, color tunable devices, induced optical heating, bio-imaging, etc^1–5^. The ZnGa_2_O_4_ is a self-activated photoluminescent material for solid state lighting^6^. The lanthanide-based ZnGa_2_O_4_ material gives large photoluminescence of the narrow band emissions^4,5^. These emissions arise due to ladder-like energy levels present in the lanthanide ions^7–11^. In various lanthanide ions, the combination of Ho^3+^/Yb^3+^ ions has been found interesting to investigate the upconversion (UC) properties in different host materials^12–15^. It has been found that this combination yields strong UC emission intensity because of energy transfer between Ho^3+^ and Yb^3+^ ions. In this case, the Yb^3+^ ion acts as sensitizer. The emission intensity of phosphor materials could also be enhanced by incorporating trace amount of some dopant ions, for example Li^+^, Mg^2+^, Zn^2+^, Ca^2+^, etc^15–17^.

The photoluminescence properties of Ho^3+^/Yb^3+^ activated phosphors were improved considerably in recent years by adding different dopant ions, which play the role of surface modifiers and sensitizers in the host materials^16,17^. The dopant ions, i.e. Li^+^, Mg^2+^, Zn^2+^, Bi^3+^ and Cr^3+^ act as surface modifiers^15–19^. These ions have modified local crystal structure around the acceptor ions for better emission intensity in the materials. Out of these, the Bi^3+^ ion has been used as surface modifier to improve the UC intensity of Er^3+^/Yb^3+^ activated La_2_O_3_ material^7^. Alternatively, the Bi^3+^ ion has also been selected as sensitizer in the downshifting (DS) process in which it transfers its energy to the Dy^3+^ and Tb^3+^ ions in the YPO_4_ and Y_2_O_3_ phosphor materials, respectively. This improves the emission intensity of the phosphor materials^20,21^. Thus, the Bi^3+^ ion is a promising material to increase the photoluminescence intensity of phosphor samples for the UC and DS processes^7,21^. The increment of UC intensity in the Ho^3+^/Yb^3+^ activated phosphor samples were observed by our group in the presence of Li^+^ and Mg^2+^ ions^15,16^. However, Kumar et al. have investigated the improvement in UC emissions of the Ho^3+^/Yb^3+^:Gd_2_O_3_ phosphor in the presence of Ca^2+^/Zn^2+^ ions^17^. Moreover, Cheng et al. have discussed UC process of the Er^3+^/Yb^3+^:ZnGa_2_O_4_ phosphor in the presence of Cr^3+^ ion^18^. The increase in emission intensity of the phosphor has been also observed through doping of Bi^3+^ ion^19^. The UC emission intensity of Ho^3+^/Yb^3+^:ZnGa_2_O_4_ material was enhanced significantly through doping of Li^+^ ion^22^. However, the emissive properties of Ho^3+^/Yb^3+^/Bi^3+^ co-doped ZnGa_2_O_4_ phosphor remains unexplored to our knowledge.

The UC emissions of phosphor materials can further be used to investigate their application in temperature sensing. The temperature sensing process is generally related to the fluorescence intensity ratio (FIR) of two close lying thermally coupled levels (i.e. TCLs)^3,4,7,8^. The range of energy gap is usually 100–2000 cm^−1^ for TCLs and it is quite different for different lanthanide ions^4,13^. These levels are affected by a small variation in external temperatures of the phosphor, which influences the intensity of emission bands originated from the TCLs^3,8,13^. The rise in temperature would increase the lattice vibrations and this leads to shift of some excited ions from a lower level to the upper level of TCLs. The temperature sensing properties have been investigated by many groups of workers in various sets of the lanthanide co-doped phosphor materials^3,4,7,8,13^. It was noticed that a change in intensity of the emission bands occurs due to increase in temperature of the phosphors. Chai et al^13^ have studied the UC-based temperature sensing in Ho^3+^/Yb^3+^:ZnWO_4_ phosphor. The intensity of green bands decreases regularly with the increase in temperature of the phosphor. However, the optical thermometry has also been reported by Kumar et al. in Ho^3+^/Yb^3+^:Gd_2_O_3_ phosphor through incorporation of Ca^2+^/Zn^2+^ ions using TCLs of Ho^3+^ ion^17^. Moreover, the temperature sensing properties was also reported by Lojpur et al. in Ho^3+^/Yb^3+^:Y_2_O_3_ phosphor^23^. As has been mentioned earlier, the UC intensity of Ho^3+^/Yb^3+^/Bi^3+^ activated ZnGa_2_O_4_ material has been not investigated. In addition to this, the temperature sensing properties is also not studied in the Ho^3+^/Yb^3+^/Bi^3+^ co-doped ZnGa_2_O_4_ phosphor to our knowledge.

Color tunability is a very fascinating property of the lanthanide ions. It occurs due to change in number of the ions in different higher energy states. The color tunability was observed not only in the downshifting (DS) but also in the UC-based phosphor materials^2,4,13,24,25^. The variations in concentration and excitation wavelength of the Tb^3+^ based DS phosphor showed color tunability^9^. The change in concentrations of the lanthanide ions in the UC-based phosphors also showed color tunability^5^. The color tunability features of Ho^3+^/Yb^3+^ activated UC phosphor samples were reported by exciting them at different power densities of 980 nm^13,25^. Our group has also reported the influence of concentrations as well as power densities on the UC intensity and obtained distinct color tunability features in the Er^3+^/Yb^3+^ based UC phosphor^4^. However, it would also be interesting to understand the influence of dopant concentration as well as power density on the color tunability of Ho^3+^/Yb^3+^/Bi^3+^ co-doped ZnGa_2_O_4_ phosphor.

In this paper, the Ho^3+^; Ho^3+^/Yb^3+^ and Ho^3+^/Yb^3+^/Bi^3+^ doped and co-doped ZnGa_2_O_4_ phosphor materials have been prepared by using solid-state reaction method. The X-ray diffraction (XRD), scanning electron microscopy (SEM) and energy dispersive X-ray spectroscopy (EDS) measurements have been used for identifying the phase formation, crystallinity, crystallite size, particles shape and size, and elemental traces present in the ZnGa_2_O_4_ materials. The Fourier transform infrared (FTIR) measurements were performed to confirm phonon energy of the phosphor lattice. The UV–vis–NIR measurements reveal the absorption characteristics of Ho^3+^/Yb^3+^/Bi^3+^ ions in the samples. The Ho^3+^ doped phosphor sample gives relatively large green emission under 980 nm excitations. The emission intensity of phosphor increased significantly through doping of Yb^3+^ and Yb^3+^/Bi^3+^ ions. The UC intensity ~ power density and lifetime measurements have also been performed to know the mechanisms of UC process and decay behaviors of the phosphor materials, respectively. The temperature sensing sensitivity has also been observed in Ho^3+^/Yb^3+^/Bi^3+^ activated ZnGa_2_O_4_ sample. The color tunability, correlated color temperature (CCT) and color purity are also discussed with the concentration of Bi^3+^ ions and power density. The highly intense UC emissions in the Ho^3+^/Yb^3+^/Bi^3+^ co-doped ZnGa_2_O_4_ phosphors can be found appropriate for the fabrication of UC based color tunable devices, green light emitting diodes (g-LEDs) and temperature sensors.

## Results and discussion

### Structural and morphological studies

#### XRD measurements

**Figure 1:**
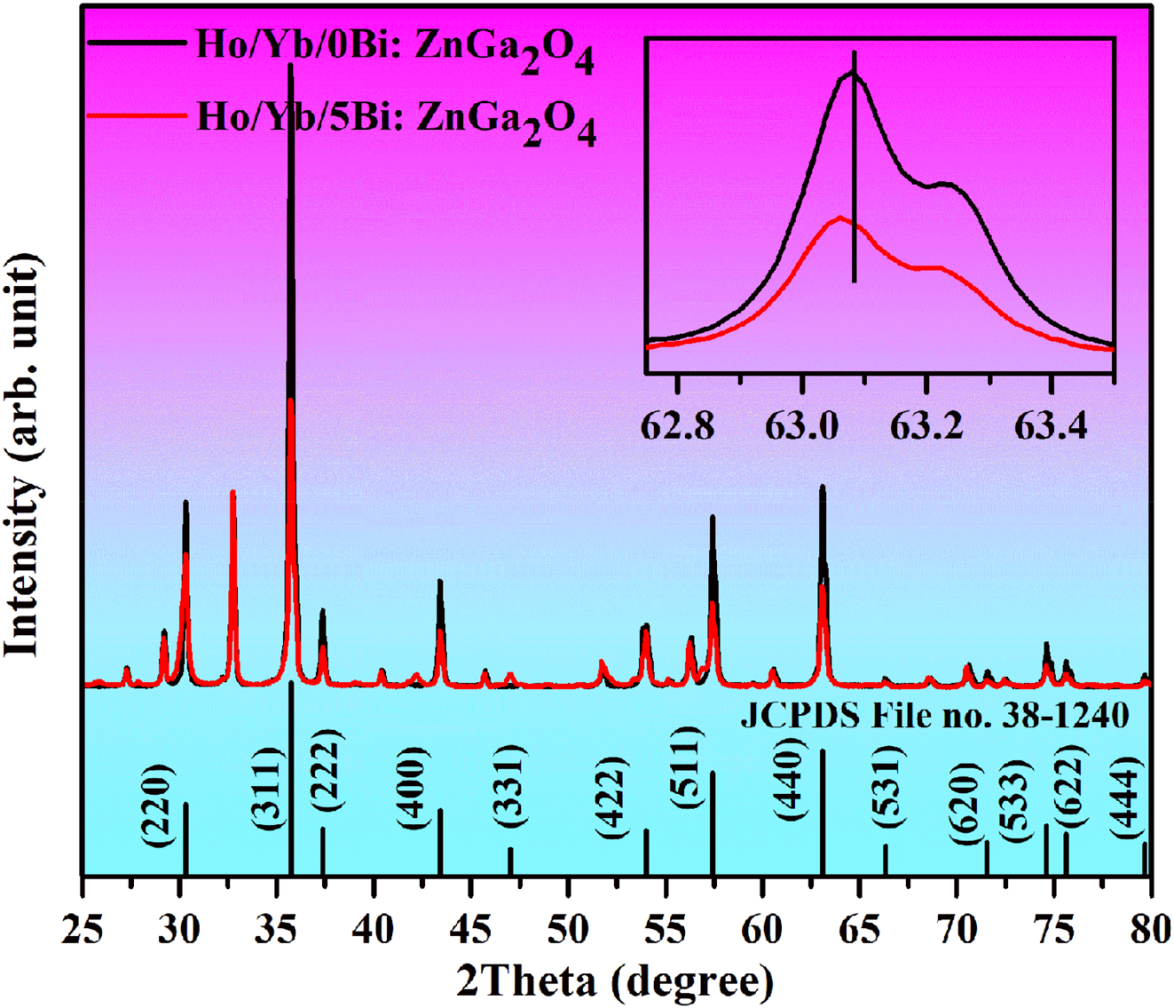
The XRD patterns of the Ho^3+^/Yb^3+^ co-doped ZnGa_2_O_4_ phosphor materials with and without the Bi^3+^ ion and the inset of Fig. 1 shows a variation of FWHM and a shift in XRD peak for 63.06° angle.

The XRD patterns of Ho^3+^/Yb^3+^/0Bi^3+^ and Ho^3+^/Yb^3+^/5Bi^3+^ co-doped ZnGa_2_O_4_ phosphors examined in the 2θ region of 25–80° angles are given in Fig. 1. The sharp and intense XRD peaks are observed in both the cases, which show the crystalline nature of phosphor samples. The XRD patterns are well matched to JCPDS File number 38–1240^4,6^. The phase of phosphor is confirmed to cubic with a space group of Fd $\overline{3}$ m(227). The cell constants for cubic phase are identified as a = b = c = 8.334 Å and α = β = γ = 90°, respectively. However, some additional XRD peaks are observed due to the Ga_5_Yb_3_O_12_ compound (JCPDS File no. 73–1373). The crystallite size of Ho^3+^/Yb^3+^/0Bi^3+^ and Ho^3+^/Yb^3+^/5Bi^3+^ co-doped materials has been calculated from Debye–Scherrer formula^21^:

i $$D = \frac{k\lambda }{{\beta cos\theta }}$$

where D refers to crystallite size; λ (= 0.15406 nm) is the X-ray radiation wavelength; β shows FWHM (full width at half maximum) of the peaks in radian; θ is diffraction angle and k (= 0.90) is the shape factor. The crystallite size values are calculated to be 31.79 and 33.38 nm for the Ho^3+^/Yb^3+^/0Bi^3+^ and Ho^3+^/Yb^3+^/5Bi^3+^ activated phosphor materials, respectively. Thus, the crystallinity of phosphor increases through Bi^3+^ doping. The increase in crystallinity can also be confirmed from the inset of Fig. 1 in which the FWHM of peak is reduced and shifted towards lower angle side through Bi^3+^ doping. This is attributed to larger ionic radius of Bi^3+^ ion (1.03 Å) compared to Zn^2+^ ion (0.74 Å). This indicates that the Bi^3+^ doping does not affect the phase of sample; however, it increases crystallinity of the Ho^3+^/Yb^3+^ co-doped sample.

The dislocation density of the Ho^3+^/Yb^3+^/0Bi^3+^ and Ho^3+^/Yb^3+^/5Bi^3+^ co-doped ZnGa_2_O_4_ materials has also been calculated by using the following relation^26^:

ii $$\delta = \frac{1}{{D^{2} }}$$

where δ is the dislocation density, which reduces with the increase of crystallite size of phosphor. The dislocation density is found to be 9.9 × 10^14^ and 8.9 × 10^14^ m^−2^ with respect to the Ho^3+^/Yb^3+^/0Bi^3+^ and Ho^3+^/Yb^3+^/5Bi^3+^ co-doped phosphor materials, respectively. This confirms that the dislocation density of phosphor decreases through Bi^3+^ doping. This also indicates an enhancement of local crystal structure around the lanthanide ions in phosphor. The microstrain (e) is also evaluated in the two phosphor materials by using the following relation^27^:

iii $$e = \frac{\beta }{4tan\theta }$$

where the terms show the usual meaning. The values of microstrain are obtained as 11.4 × 10^−2^ and 10.8 × 10^−2^ for the Ho^3+^/Yb^3+^/0Bi^3+^ and Ho^3+^/Yb^3+^/5Bi^3+^ co-doped phosphor materials, respectively. It shows that the microstrain of phosphor reduces via doping of Bi^3+^ ion. Therefore, the XRD analyses elaborate that not only growth in crystallinity but also a decrease in dislocation density and microstain would be supportive for getting large UC intensity from the phosphor materials.

### SEM and EDS analyses

**Figure 2:**
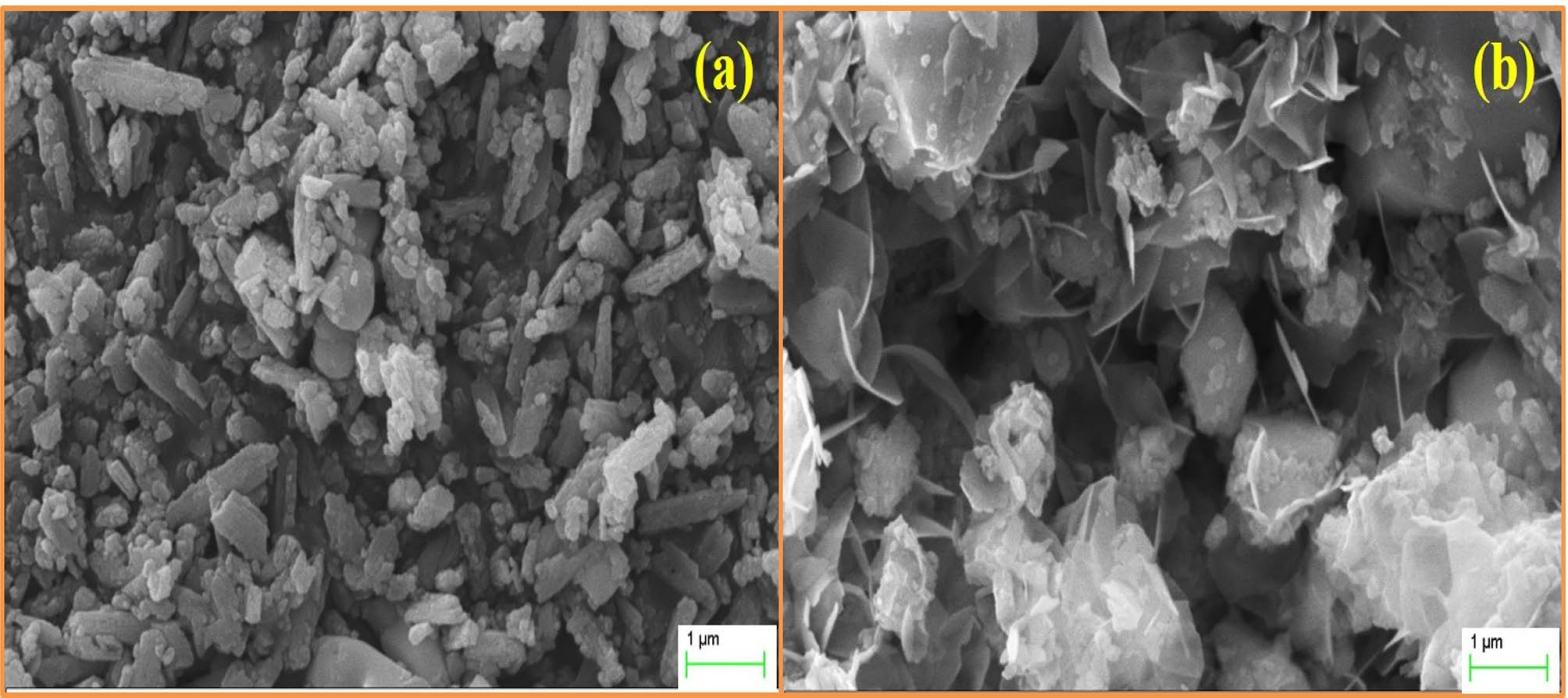
The SEM images of (**a**) Ho^3+^/Yb^3+^/0Bi^3+^ and (**b**) Ho^3+^/Yb^3+^/5Bi^3+^ co-doped phosphor materials.

Figure 2 represents the SEM images of the Ho^3+^/Yb^3+^/0Bi^3+^ and Ho^3+^/Yb^3+^/5Bi^3+^ co-doped ZnGa_2_O_4_ phosphor materials. The particles of phosphors are found in random manners with the agglomerated features. The particles shape of phosphor is changed to the flower-like structure through Bi^3+^ doping. However, the particles size of phosphor material is observed to increase (see Fig. 2b). The change in particles shape and size of different host materials has been discussed by the other researchers in the presence of different surfactants and dopant ions^28,29^. The formation of larger sized particles was also observed by our group and Wu et al. in the Er^3+^/Yb^3+^:La_2_O_3_ and Er^3+^:Y_2_O_2_S phosphor materials, respectively through Bi^3+^ doping^7,30^. In our case, the average value of particles size is obtained as 0.70 µm for the Ho^3+^/Yb^3+^ co-doped phosphor and it increases to 0.82 µm through Bi^3+^ doping. Thus, the particles shape and size of phosphor are modified through Bi^3+^ doping.

**Figure 3:**
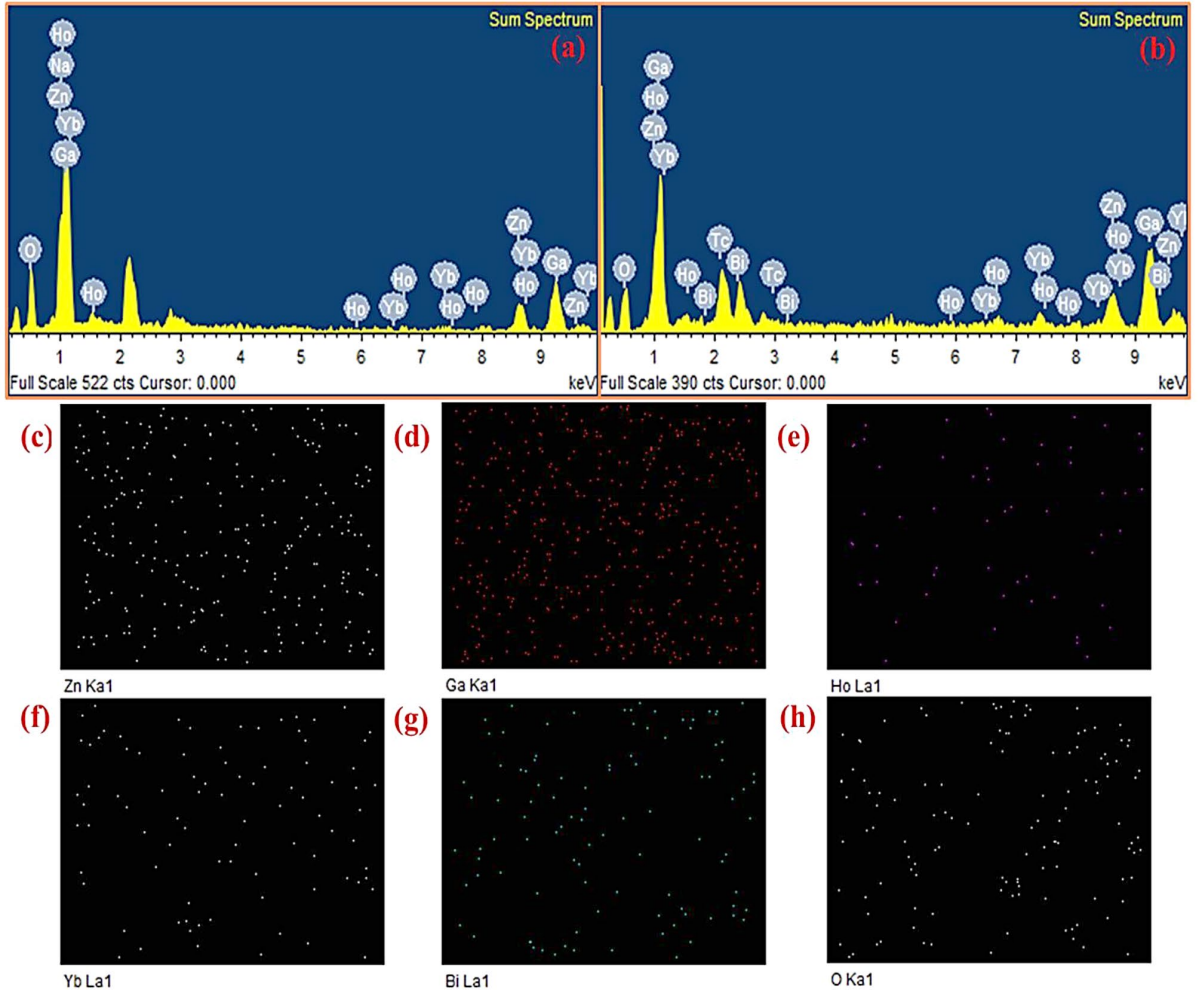
(**a**, **b**) The EDS spectra of the Ho^3+^/Yb^3+^/0Bi^3+^ and Ho^3+^/Yb^3+^/5Bi^3+^ co-doped phosphors. (**c**–**h**) represent the EDS mappings of Zn, Ga, Ho, Yb, Bi and O constituents in the Ho^3+^/Yb^3+^/5Bi^3+^ co-doped phosphor generated by using INCA software.

Figure 3a,b depict the EDS spectra of Ho^3+^/Yb^3+^/0Bi^3+^ and Ho^3+^/Yb^3+^/5Bi^3+^ co-doped ZnGa_2_O_4_ phosphor materials. The spectra reveal that the phosphor materials contain Bi, Ga, Ho, O, Yb and Zn elements. Figure 3c,h show the EDS mappings of the Zn, Ga, Ho, Yb, Bi and O constituents in the Ho^3+^/Yb^3+^/5Bi^3+^ co-doped material generated by using INCA software. These figures suggest that all the constituents are distributed almost uniformly in the phosphor sample. The distribution of these elements in the phosphor sample would be more suitable for getting larger UC emission intensity.

### Optical measurements

#### FTIR studies

**Figure 4:**
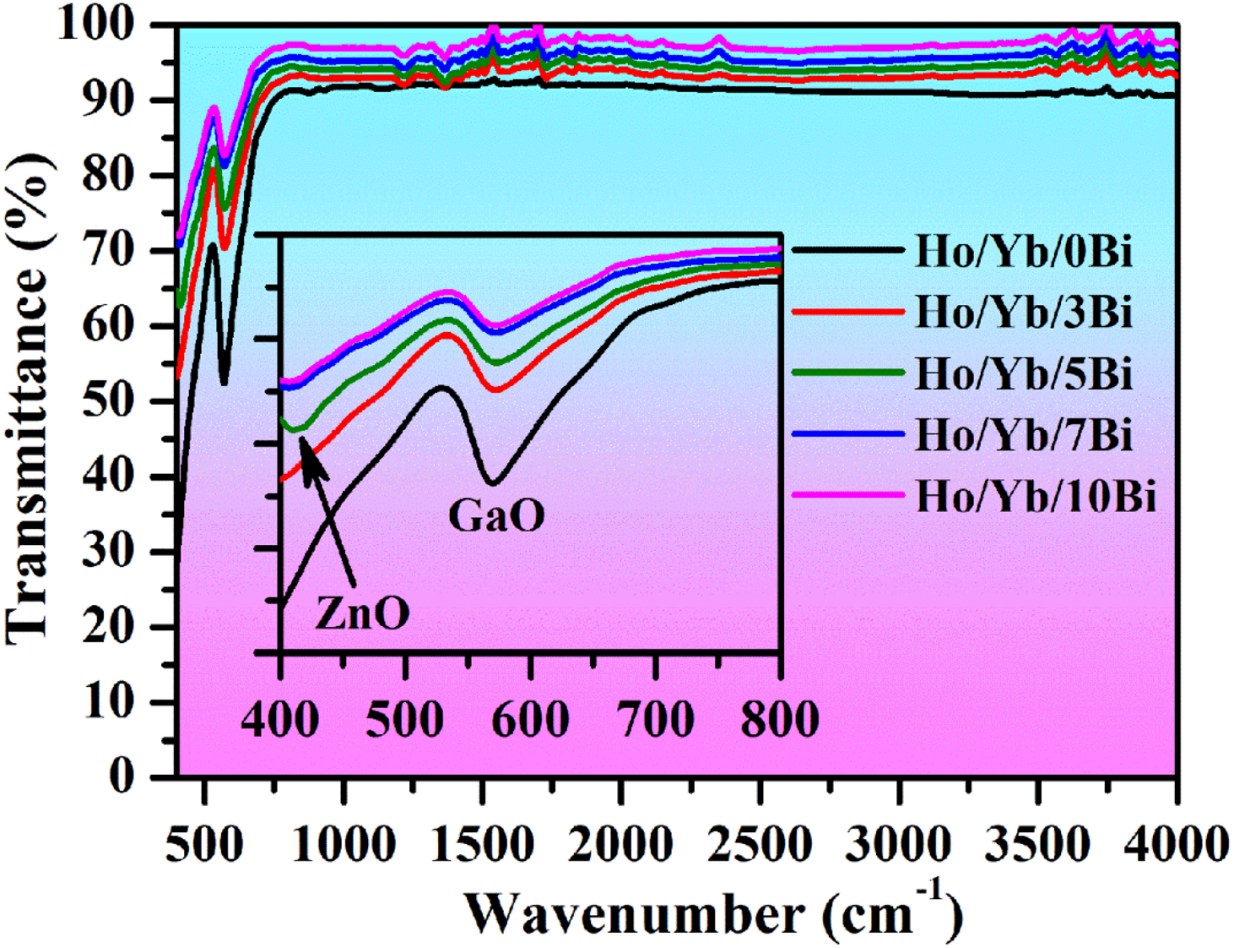
The FTIR spectra of the Ho^3+^/Yb^3+^/xBi^3+^ (i.e. x = 0, 3, 5, 7 and 10 mol%) co-doped phosphor materials.

The molecular vibrational groups existing in the phosphor materials have been studied by the FTIR measurements. The FTIR spectra of Ho^3+^/Yb^3+^/xBi^3+^ (i.e. x = 0, 3, 5, 7 and 10 mol%) co-doped ZnGa_2_O_4_ materials have been monitored in 400–4000 cm^−1^ range as revealed in Fig. 4. The vibrational frequencies are observed at 413 and 569 cm^−1^ corresponding to the stretching modes of the ZnO and GaO groups, respectively^4,6^. The position of different bands remains unchanged through Bi^3+^ doping; however, the intensity of these bands varies accordingly. Since the phosphor sample was prepared at higher temperature (at 1200 °C) the impurity peaks, such as OH^−^ and CO_3_^2−^ groups, etc. do not appear in the spectra^4^. The figure also indicates that the phonon frequency of ZnGa_2_O_4_ host is small and hence, the photoluminescence intensity of radiative transitions would be large in the phosphor materials.

#### UV–vis–NIR studies

**Figure 5:**
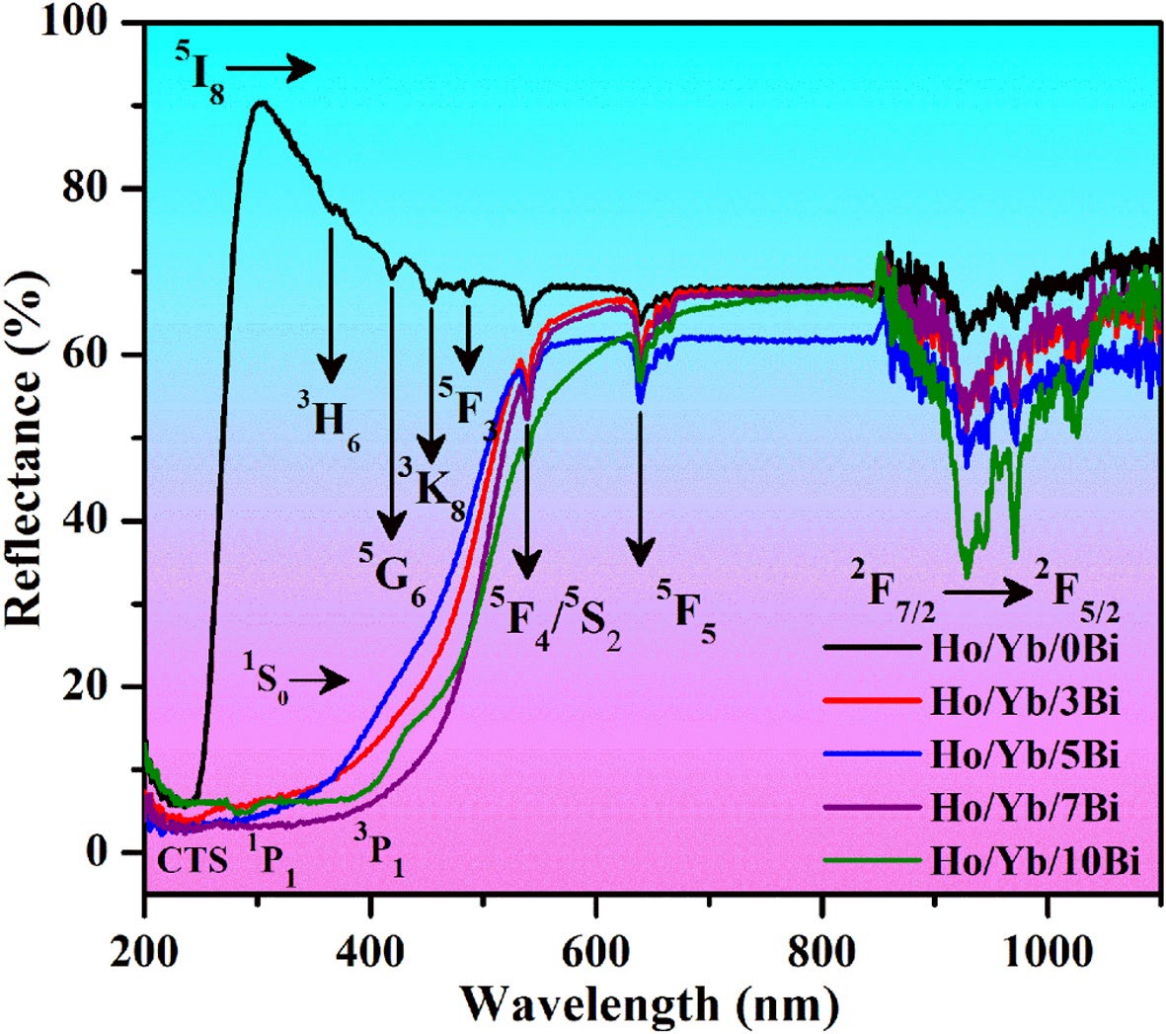
The UV–vis–NIR absorption spectra of Ho^3+^/Yb^3+^/xBi^3+^ (i.e. x = 0, 3, 5, 7 and 10 mol%) co-doped materials.

Figure 5 illustrates the UV–vis-NIR absorption spectra of the Ho^3+^/Yb^3+^/xBi^3+^ (i.e. x = 0, 3, 5, 7 and 10 mol%) co-doped phosphor materials monitored in 200–1100 nm range using diffuse reflectance mode. The band at 240 nm has been assigned to the charge transfer state (CTS) of O^2−^  → Ga^3+^ corresponding to ZnGa_2_O_4_ host^4^. Alongwith this, the spectra have different absorption peaks positioned at 366, 419, 454, 487, 540 and 639 nm because of various transitions of the Ho^3+^ ions, which are attributed through absorption from ground state (^5^I_8_) to higher excited states, such as ^3^H_6_, ^5^G_6_, ^3^K_8_, ^5^F_3_, (^5^F_4_/^5^S_2_) and ^5^F_5_, respectively^16^. After doping the Bi^3+^ ion in phosphor, a broad absorption band is also observed in the 230–405 nm region and it is the overlapped profiles of CTS of O^2−^ → Ga^3+^ ions and the absorption from ^1^S_0_ level to ^1^P_1_ (at 283 nm) and ^3^P_1_ (at 401 nm) levels of the Bi^3+^ ions^7,21^.

An intense broad absorption band has also been found at 971 nm because of ^2^F_7/2_ → ^2^F_5/2_ transition of the Yb^3+^ ion^16^. The absorption cross-section of NIR region is improved considerably due to increase in crystallinity of the phosphor through Bi^3+^ doping, which is favorable for the large excitation and radiative transitions. Since the absorption band of Yb^3+^ ion is very broad it can absorb large number of incident photons, which would generate the large UC intensity of Ho^3+^ ion.

### Optical band gap analysis

**Figure 6:**
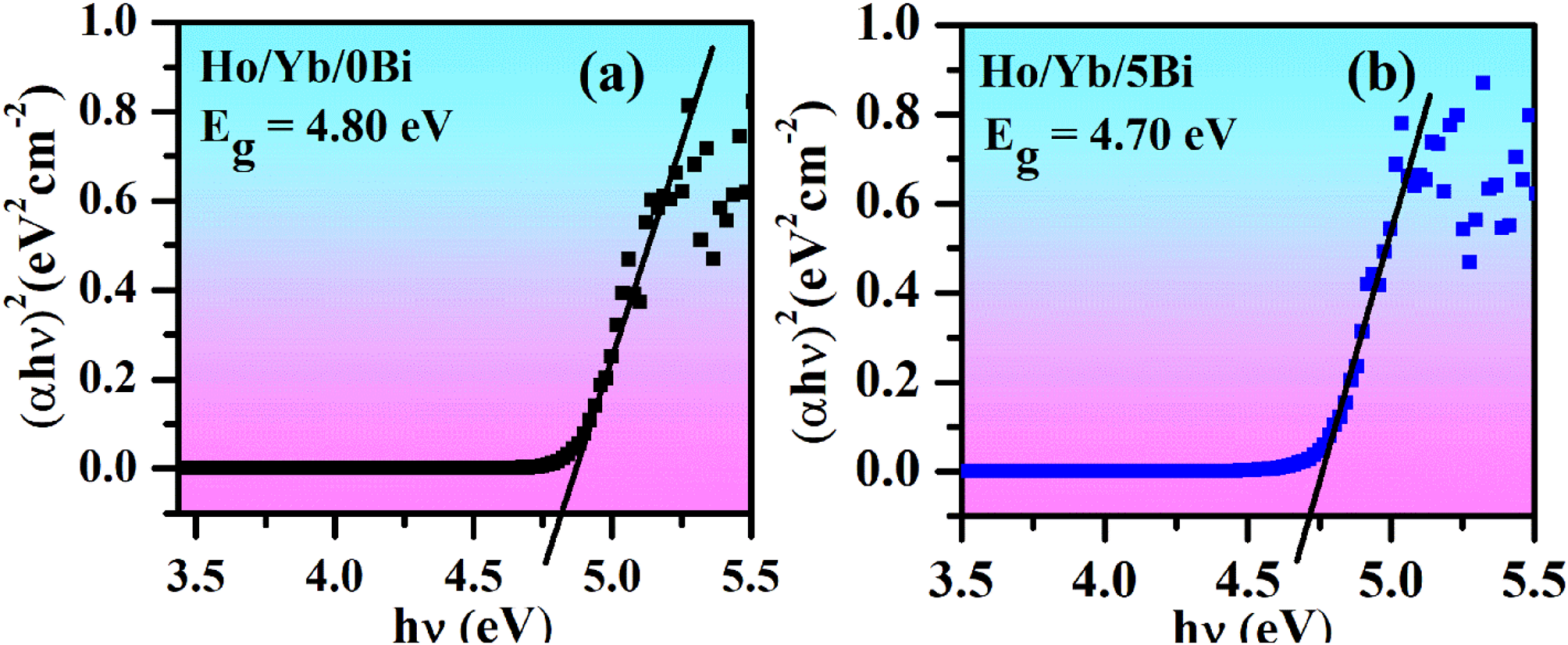
The plots between hν ~ (αhν)^2^ for the (a) Ho^3+^/Yb^3+^/0Bi^3+^ and (b) Ho^3+^/Yb^3+^/5Bi^3+^ co-doped phosphor materials.

Optical band gap of Ho^3+^/Yb^3+^/0Bi^3+^ and Ho^3+^/Yb^3+^/5Bi^3+^ co-doped ZnGa_2_O_4_ materials can be estimated by using Wood–Tauc formula^31^:

iv $$\left( {\upalpha {\text{h}}\upnu } \right)^{{1/{\text{n}}}} = {\text{ A}}\left( {{\text{h}}\upnu - {\text{E}}_{{\text{g}}} } \right)$$

where E_g_ refers to the band gap energy; hυ is the energy of incident photons; α is absorption coefficient and B is band tailoring constant. The ‘n’ value was chosen as (1/2) for the direct allowed transitions. The plotted graphs of hυ ~ (αhυ)^2^ for the Ho^3+^/Yb^3+^/0Bi^3+^ and Ho^3+^/Yb^3+^/5Bi^3+^ activated phosphors are given in Fig. 6. The values of band gap energy (E_g_) are obtained as 4.80 and 4.70 eV for the Ho^3+^/Yb^3+^/0Bi^3+^ and Ho^3+^/Yb^3+^/5Bi^3+^ activated phosphor materials, respectively.

This means that the band gap of Ho^3+^/Yb^3+^ co-doped ZnGa_2_O_4_ material decreases via Bi^3+^ doping^21^. It has been mentioned above that not only the crystallite size but the particles size of phosphor is also improved via Bi^3+^ doping. An increase in particles size will also reduce the gap between the valence and the conduction bands in ZnGa_2_O_4_ lattice. If the band gap of phosphor reduces; the large numbers of the excited ions will be transferred to the higher energy states, which would generate better UC intensity for the ZnGa_2_O_4_ materials.

### Upconversion studies

**Figure 7:**
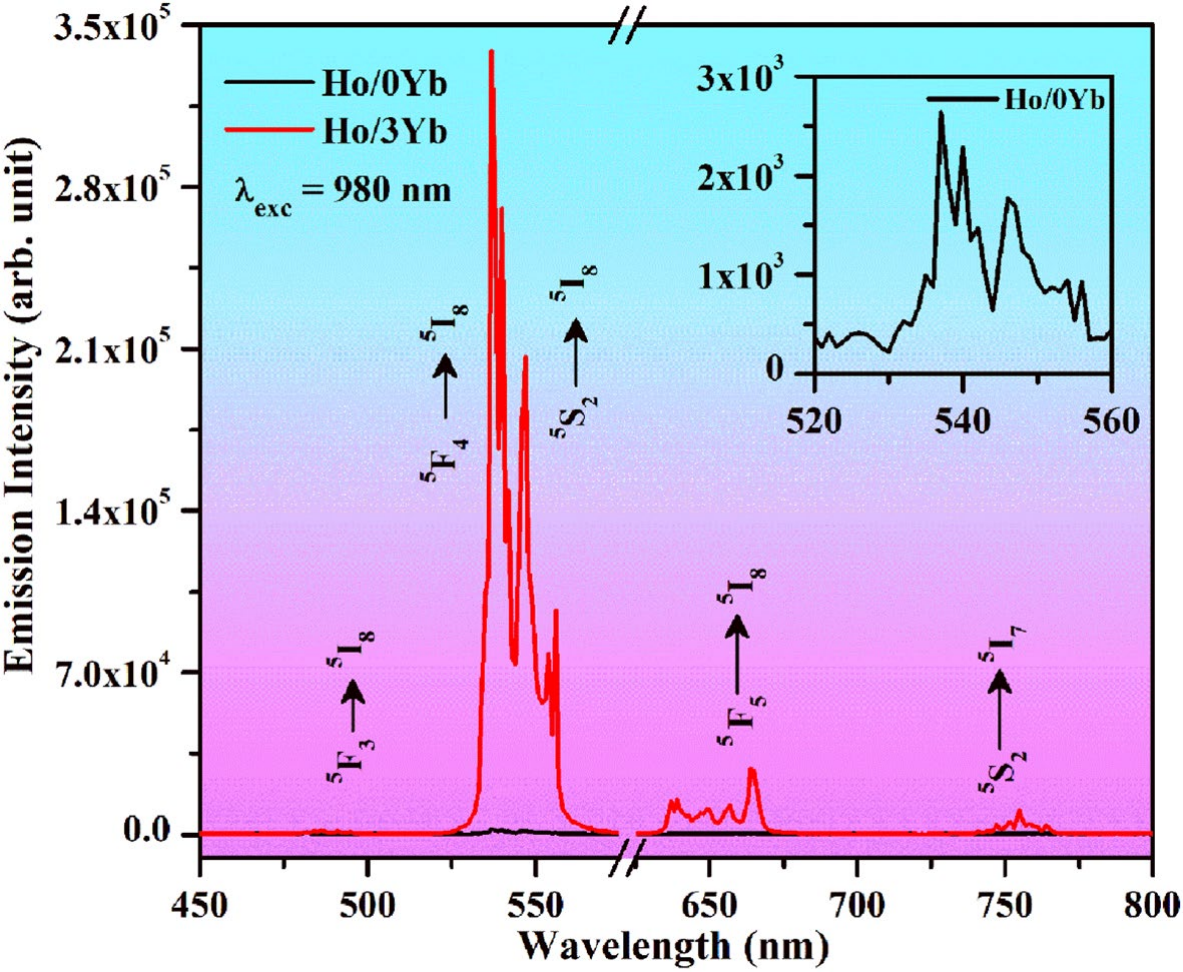
The upconversion emission spectra of Ho^3+^/0Yb^3+^ and Ho^3+^/3Yb^3+^ doped and co-doped ZnGa_2_O_4_ materials under 980 nm excitations at 31.84 W/cm^2^. The inset figure shows the enlarged emission spectrum of Ho^3+^/0Yb^3+^ doped phosphor for the green region.

The upconversion emission spectra of the Ho^3+^/0Yb^3+^ and Ho^3+^/3Yb^3+^ doped and co-doped ZnGa_2_O_4_ materials monitored in 450–800 nm range under 980 nm excitations at 31.84 W/cm^2^ are revealed in Fig. 7. In the figure, the emission spectra possess several emission peaks in the blue, green, red and NIR regions. They are centered at 486, (537, 538), 547, 664 and 755 nm and attributed to ^5^F_3_ → ^5^I_8_, ^5^F_4_ → ^5^I_8_, ^5^S_2_ → ^5^I_8_, ^5^F_5_ → ^5^I_8_ and ^5^S_2_ → ^5^I_7_ transitions of the Ho^3+^ ion, respectively^12,32–35^. The Ho^3+^ doped phosphor produces weak transitions in the green and the red regions^22^. The UC intensity of the green emission is many times higher than the red emission. However, the blue as well as NIR emissions are not found due to lack of excitation. Dey et al^33^ have also reported similar type of emissions in the green, red and NIR regions for Ho^3+^ doped CaMoO_4_ phosphor. The inset of Fig. 7 shows an enlarged spectrum of Ho^3+^ doped ZnGa_2_O_4_ phosphor for the green region.

On the other hand, these emissions could appear through doping of Yb^3+^ ion alongwith Ho^3+^ ion in the ZnGa_2_O_4_ host. The Ho^3+^ and Yb^3+^ co-doped phosphor leads to distinct appearance of not only the green and red emissions but also the blue and NIR emissions. The UC intensity of Ho^3+^ doped phosphor is improved many times in presence of Yb^3+^ ion. This attributes to the energy transfer (ET) from Yb^3+^ to Ho^3+^ ions^12–17,33,35^. The emission intensity of green color is several times stronger than the blue, red and NIR emissions. Chen et al. have also found the similar trend of UC intensity for these emissions in the SrF_2_:Gd^3+^/Yb^3+^/Er^3+^ nanocrystals^36^. Further, the emission intensity of red band is larger than the blue and NIR bands. Hence, the UC intensity of Ho^3+^ doped phosphor is increased by 128, 67 and 21 times in the presence of Yb^3+^ ion for the green, red and NIR emissions, respectively. The Yb^3+^ ion, thereby acts as sensitizer for the Ho^3+^ doped phosphor.

### Power density dependent studies

**Figure 8:**
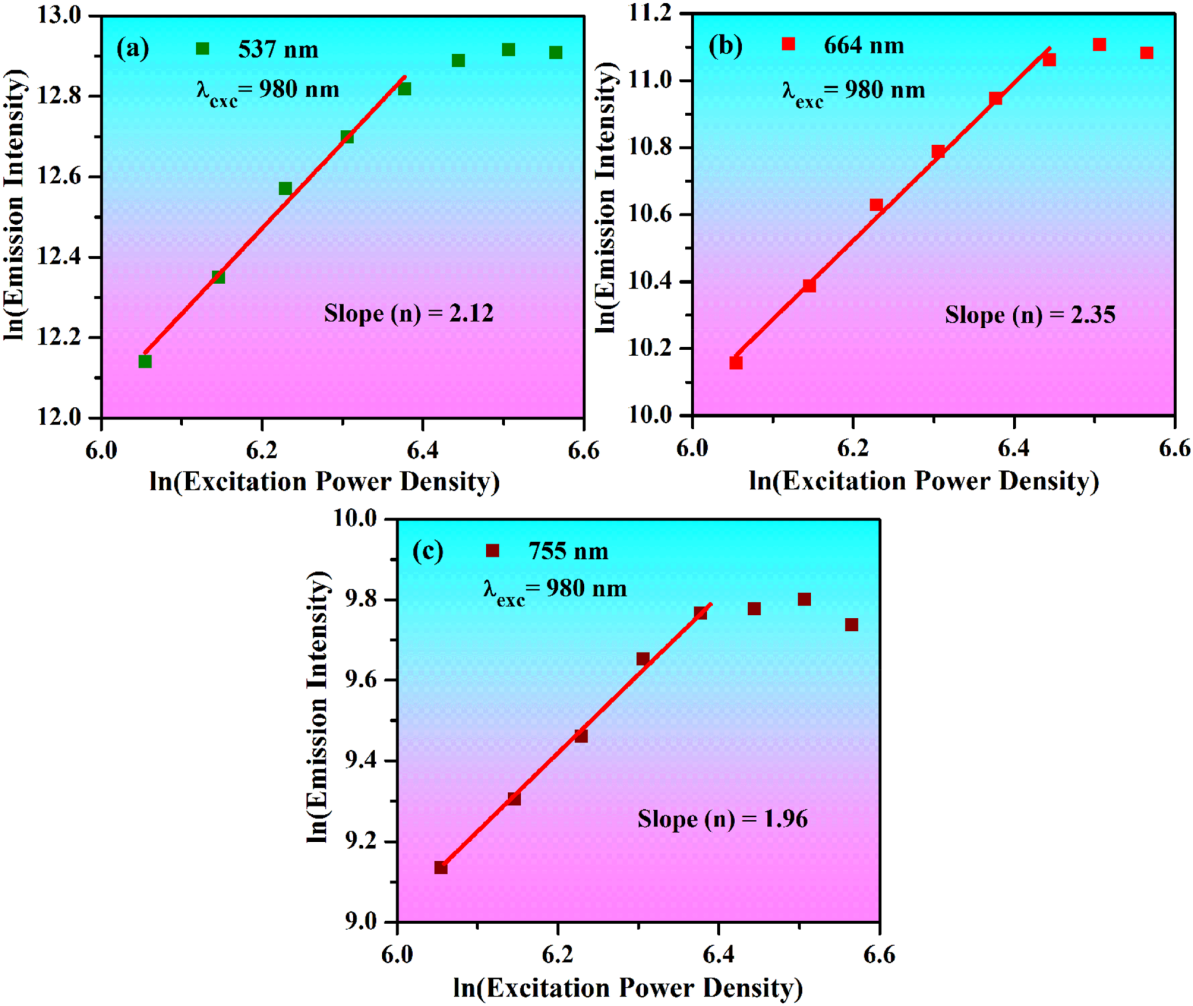
The dual logarithmic plots between the emission intensity ~ excitation power density (W/cm^2^) of (**a**) green; (**b**) red and (**c**) NIR emissions for the Ho^3+^/Yb^3+^ doped material.

The UC intensity of the Ho^3+^/Yb^3+^ co-doped ZnGa_2_O_4_ phosphor material has been monitored at various excitation power densities of 980 nm radiation. The UC emission is a nonlinear process and it is directly related to (nth) power of incident radiation^8,37^ e.g.

v $$I_{up} \upalpha P^{n} ,$$

where n indicates the number of photons participating in the UC emission, *I*_*up*_ is the upconversion intensity and *P* is excitation power density in W/cm^2^. The dual logarithmic plots between the emission intensity ~ excitation power density for the green, red and NIR emissions of the Ho^3+^/Yb^3+^ co-doped material are given in Fig. 8. The emission intensity varies linearly with excitation power density upto certain limit and saturates due to involvement of non-radiative channels at higher excitation power density. The slope values (n) have been evaluated by linear fittings of dual logarithmic plots. These values are found to be 2.12, 2.35 and 1.96 for the green, red, and NIR emissions, respectively. From this, it has been noted that the ^5^F_4_ (green), ^5^F_5_ (red) and ^5^S_2_ (NIR) levels are populated by the absorption of two photons^15^. The deviation in an integer value occurs because of non-radiative processes engaged for populating these levels. The mechanisms involved for these transitions can be discussed by using energy level diagrams of the Ho^3+^ and Yb^3+^ ions.

**Figure 9:**
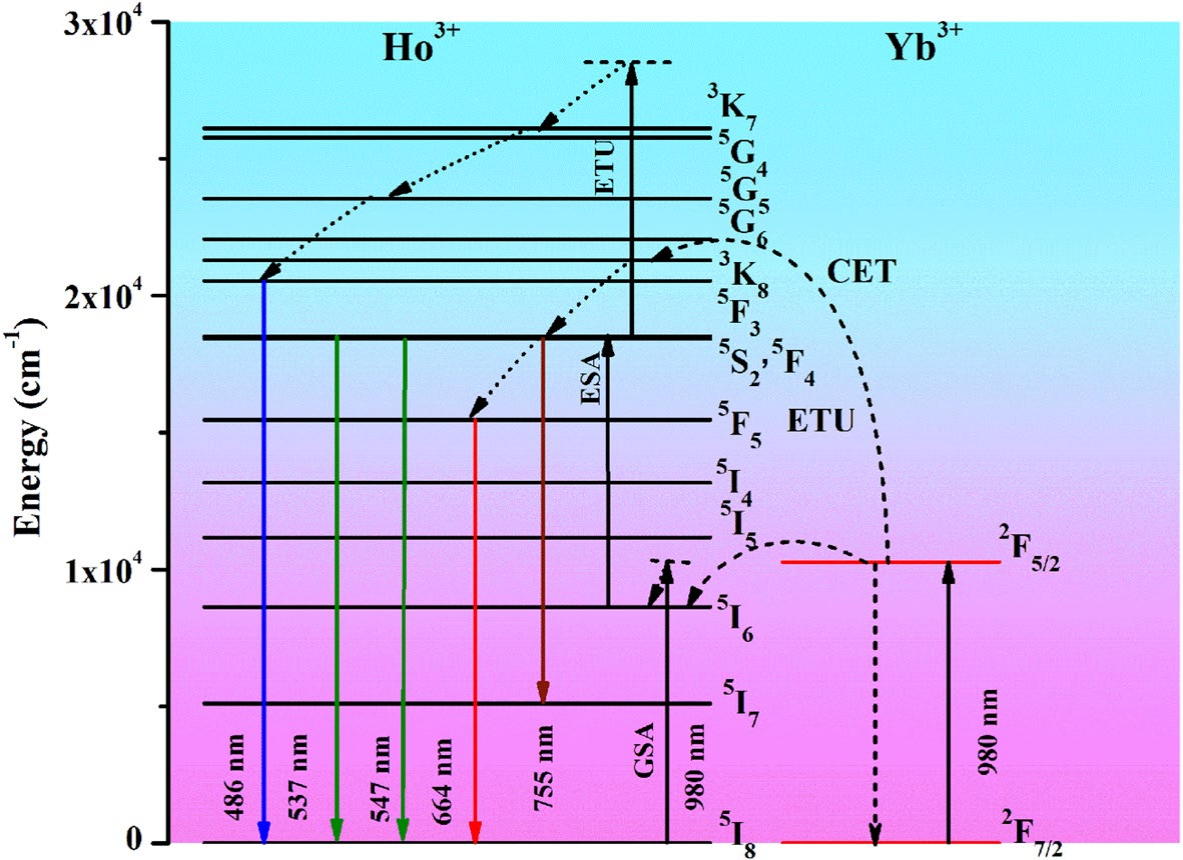
The energy level diagrams of Ho^3+^ and Yb^3+^ ions along with upconversion mechanism.

Figure 9 represents the distinct energy level diagrams of the Ho^3+^ and Yb^3+^ ions. When the Ho^3+^ doped ZnGa_2_O_4_ sample is excited with 980 nm photons it absorbs this radiation weakly either through phonon assisted excitation or via collision or both; because the Ho^3+^ ion has no resonant energy level with respect to 980 nm radiation. Due to this, a small number of the ions are shifted from ground state (^5^I_8_) to higher state (^5^I_6_) via ground state absorption (GSA) process. The Ho^3+^ ion present in ^5^I_6_ level reabsorbs 980 nm radiations and thereby populated the (^5^F_4_/^5^S_2_) excited states via excited state absorption (ESA) process. The excited ions in these states produce weak radiation in the green region^22,33^. Some ions are relaxed non-radiatively to populate the ^5^F_5_ state and due to this, a very weak red emission takes place. However, the emissions in the blue and NIR regions are not clearly identified due to lack of excitation. These emissions are clearly detected in the presence of Yb^3+^ ion (see Fig. 7).

The Yb^3+^ sensitizer ions transfer its excitation energy to Ho^3+^ ions via cooperative energy transfer (CET) and energy transfer upconversion (ETU) processes as can be seen from Fig. 9. When the Yb^3+^ and Ho^3+^ ions are added together in the ZnGa_2_O_4_ material, it gives strong emission because of ET from Yb^3+^ to Ho^3+^ ions. Actually, the excited level of Yb^3+^ ion is well matched with 980 nm radiation^4,11^. Therefore, on exciting the Ho^3+^/Yb^3+^ co-doped ZnGa_2_O_4_ sample by 980 nm diode laser the Yb^3+^ ions are promoted to its excited state (^2^F_5/2_) from the ground state (^2^F_7/2_). The Yb^3+^ ions thus transfer their excitation energy to the Ho^3+^ ions via ETU/CET processes, which promote them to different excited states, i.e. ^5^I_6_, ^3^K_8_ and ^3^K_7_ states. Thus, the population of Ho^3+^ ions in ^5^I_6_ state is increased enormously through GSA/ETU processes. The ions in the ^5^I_6_ state reabsorb 980 nm radiations and they jumped to (^5^F_4_/^5^S_2_) excited states by ESA/ETU processes. These excited states are further populated by the non-radiative transitions of ions from ^3^K_8_ state because ^3^K_8_ state is populated by ETU and CET processes. In CET process, two Yb^3+^ ions in the excited state combine together and transfer its energy to Ho^3+^ ions simultaneously^12–17^. Thus, the excited (^5^F_4_/^5^S_2_) states are populated with huge number of the Ho^3+^ ions and they return to the ground state by emitting strong green emission peaks at 537 and 547 nm. The weak NIR emission also occurs at 755 nm from ^5^S_2_ state to ^5^I_7_ state transition. Some of the ions present in (^5^F_4_/^5^S_2_) states are relaxed non-radiatively to ^5^F_5_ state. Due to this, a relatively weak red emission has been observed at 664 nm because of ^5^F_5_ → ^5^I_8_ transition. Finally, the ^3^K_7_ level is populated through ETU process and these ions relaxed non-radiatively to ^5^F_3_ level. The ions present in ^5^F_3_ level produce weak blue emission at 486 nm. Thus, the blue, green, red and NIR emissions are detected distinctly due to absorption of three/two NIR photons in different excited states^32–35^.

### Effect of Bi^3+^ doping

**Figure 10:**
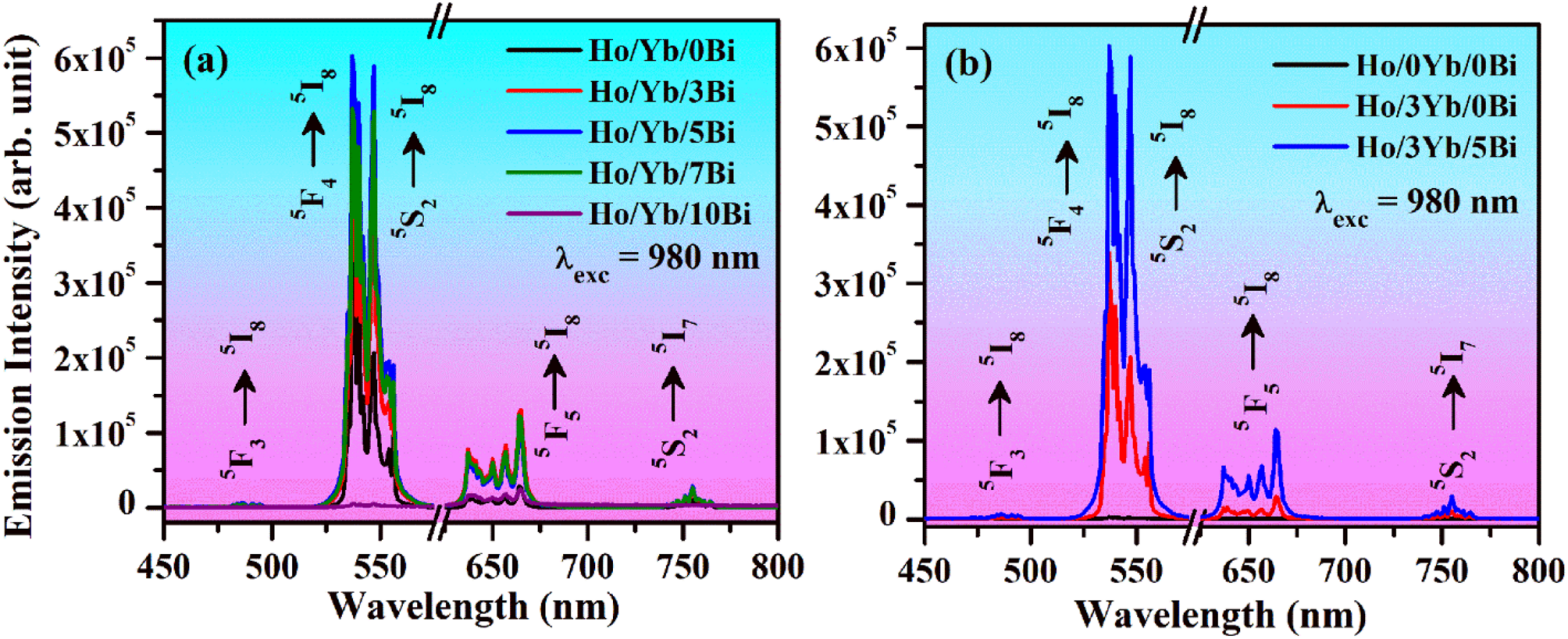
(**a**) The UC spectra of the Ho^3+^/Yb^3+^/xBi^3+^ (i.e. x = 0, 3, 5, 7 and 10 mol%) co-doped phosphors monitored under 980 nm excitations at 31.84 W/cm^2^. (**b**) The comparison of UC emission intensities of Ho^3+^ doped, Ho^3+^/Yb^3+^ and Ho^3+^/Yb^3+^/5Bi^3+^ co-doped phosphors monitored under 980 nm excitations at 31.84 W/cm^2^.

As have been mentioned earlier, the Bi^3+^ ion is a very effective dopant, which has often been used as sensitizer as well as the surface modifier^7,19^. In order to understand the impact of Bi^3+^ ion on the UC intensity of Ho^3+^/Yb^3+^ co-doped samples, we have prepared the phosphors with various concentrations of Bi^3+^ ion (i.e. 3, 5, 7 and 10 mol%) and monitored their UC emission intensity in 450–800 nm range under 980 nm excitations at 31.84 W/cm^2^ power density. It has been noticed that the UC intensity of the samples enhances appreciably. Figure 10a depicts the UC emission spectra of Ho^3+^/Yb^3+^/xBi^3+^ (i.e. x = 0, 3, 5, 7 and 10 mol%) co-doped phosphors monitored under 980 nm excitations. The UC emission peaks observed via Bi^3+^ doping is similar to those observed in Ho^3+^/Yb^3+^ co-doped ZnGa_2_O_4_ material. However, the UC intensity of emission peaks is improved by several times. Firstly, the emission intensity is observed to enhance for 3 and 5 mol% concentrations of Bi^3+^ ion and it is larger for 5 mol% concentrations. The further increase in the concentrations of Bi^3+^ ion tends to a decrement in the UC intensity (i.e. for 7 and 10 mol%) due to concentration quenching. In this process, the excitation energy is lost in terms of multi-polar interactions because of a shorter gap among the Ho^3+^/Yb^3+^ ions than their critical distance^8,15,33^. The similar observation has also been found by Li et al^38^ in the Sr_2_P_2_O_7_:Bi^2+^ material. The effect of Bi^3+^ doping was also studied by Xu et al^39^ in the Sm^3+^/Eu^3+^ coactivated Ca_20_Al_26_Mg_3_Si_3_O_68_ phosphor in which the concentration quenching also takes place at higher concentrations of Bi^3+^ ions. Our group has also studied the impact of Bi^3+^ ion on the UC intensity of Er^3+^/Yb^3+^ activated phosphors and observed concentration quenching after 5 mol% concentrations of Bi^3+^ ions^7,19^. Wang et al^40^ have also reported concentration quenching in the NaGdF_4_:2%Er^3+^ phosphor after 25 mol% concentrations of Ca^2+^ ions. In the present case, we have also observed concentration quenching above 5 mol% concentrations of Bi^3+^ ion. Therefore, the UC intensity of Ho^3+^/Yb^3+^ co-doped ZnGa_2_O_4_ phosphor material is optimum at 5 mol% concentration of Bi^3+^ ion.

Figure 10b shows the comparison of emission intensities between Ho^3+^ doped, Ho^3+^/Yb^3+^ and Ho^3+^/Yb^3+^/5Bi^3+^ co-doped phosphors under 980 nm excitations at 31.84 W/cm^2^. As discussed above, the UC intensity of Ho^3+^ doped phosphor is increased by 128, 67 and 21 times for the green, red and NIR emission bands through Yb^3+^ doping. This arises because of ET from Yb^3+^ to Ho^3+^ ions^41^. Moreover, we have again observed an improvement in the UC intensity by 228, 272 and 57.7 times for the green, red and NIR emission peaks, respectively through Yb^3+^/Bi^3+^ co-doping compared to the pure Ho^3+^ doped material. Similarly, the UC intensity of Yb^3+^/Er^3+^ activated Zn_2_SiO_4_ material was improved by several times via Bi^3+^ doping^42^. It means that the Bi^3+^ doping helps significantly to improve the UC intensity of different materials.

The large enhancement in UC intensity has been discussed by taking accounts of several important parameters through Bi^3+^ doping. The improvement in crystallite size from 31.79 to 33.38 nm, decrease in dislocation density from 9.9 × 10^14^ to 8.9 × 10^14^ m^−2^ and microstrain from 11.4 × 10^−2^ to 10.8 × 10^−2^ through Bi^3+^ doping has created a large crystalline structure around the Ho^3+^ and Yb^3+^ ions. The particles size of phosphor is relatively larger through Bi^3+^ doping (see Fig. 2b). The band gap energy of phosphor is also decreased through Bi^3+^ doping, which improves the rate of excitation of the ions from the ground state to the higher energy states because of smaller gap between valence and conduction bands (see Fig. 6b). This would be responsible for generating large UC intensity in the Ho^3+^/Yb^3+^ co-doped ZnGa_2_O_4_ phosphor. The intensity of vibrational bands of ZnGa_2_O_4_ phosphor also varies in the presence of Bi^3+^ ion. The absorption cross-section of NIR region is also improved considerably due to increase in crystallinity of the phosphor through Bi^3+^ doping, which is favorable for the large excitation and radiative transitions^7,19^. All these parameters together played an essential role for large enhancement in UC intensity of the phosphor through doping of Bi^3+^ ion.

### Lifetime studies

**Figure 11:**
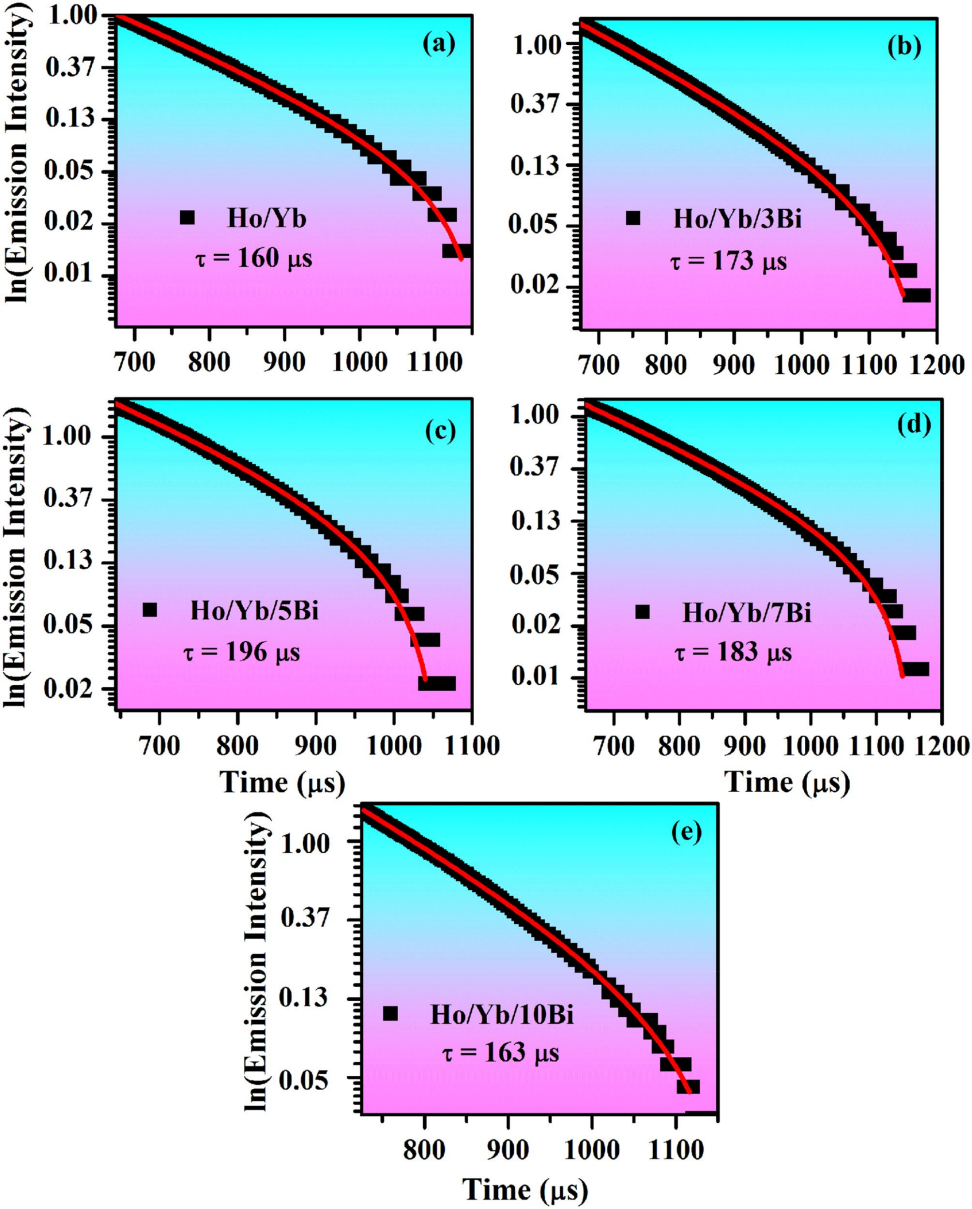
Decay curves of the ^5^F_4_ level of Ho^3+^/Yb^3+^/xBi^3+^ co-doped ZnGa_2_O_4_ materials under 980 nm excitation at 31.84 W/cm^2^, i.e. x = (**a**) 0 mol%, (**b**) 3 mol%, (**c**) 5 mol%, (**d**) 7 mol% and (**e**) 10 mol%.

The lifetime of ^5^F_4_ level in the Ho^3+^/Yb^3+^/xBi^3+^ (x = 0, 3, 5, 7 and 10 mol%) co-doped phosphors has been monitored by exciting them at 980 nm using 31.84 W/cm^2^ power density. The decay curve of Ho^3+^/Yb^3+^/xBi^3+^ co-doped materials are given in Fig. 11. These decay curves were fitted mono-exponentially according to the following relation^4,16^:

vi $$I = I_{0} e^{ - t/\tau }$$

where I_0_ and I refer to the initial and final emission intensity for 0 and t times, respectively. The term (τ) refers to lifetime of the ^5^F_4_ level.

The values of lifetime have been calculated and found to be 160, 173, 196, 183 and 163 μs for the Ho^3+^/Yb^3+^/xBi^3+^ (i.e. x = 0, 3, 5, 7 and 10 mol%) co-doped materials, respectively. It is clear from Fig. 11 that the decay time of ^5^F_4_ level of the Ho^3+^ ion increases through Bi^3+^ doping^16,19,21^. This supports an increase in UC intensity generated from the phosphor sample. The lifetime value increases upto 5 mol% then found to decrease at higher concentrations (i.e. 7 and 10 mol%). The lifetime value is expected to increase because of improvement in local crystal structure around the Ho^3+^ and Yb^3+^ ions in the phosphor^16^.

### Temperature sensing sensitivity in Ho^3+^/Yb^3+^/5Bi^3+^ co-doped ZnGa_2_O_4_ phosphor

**Figure 12:**
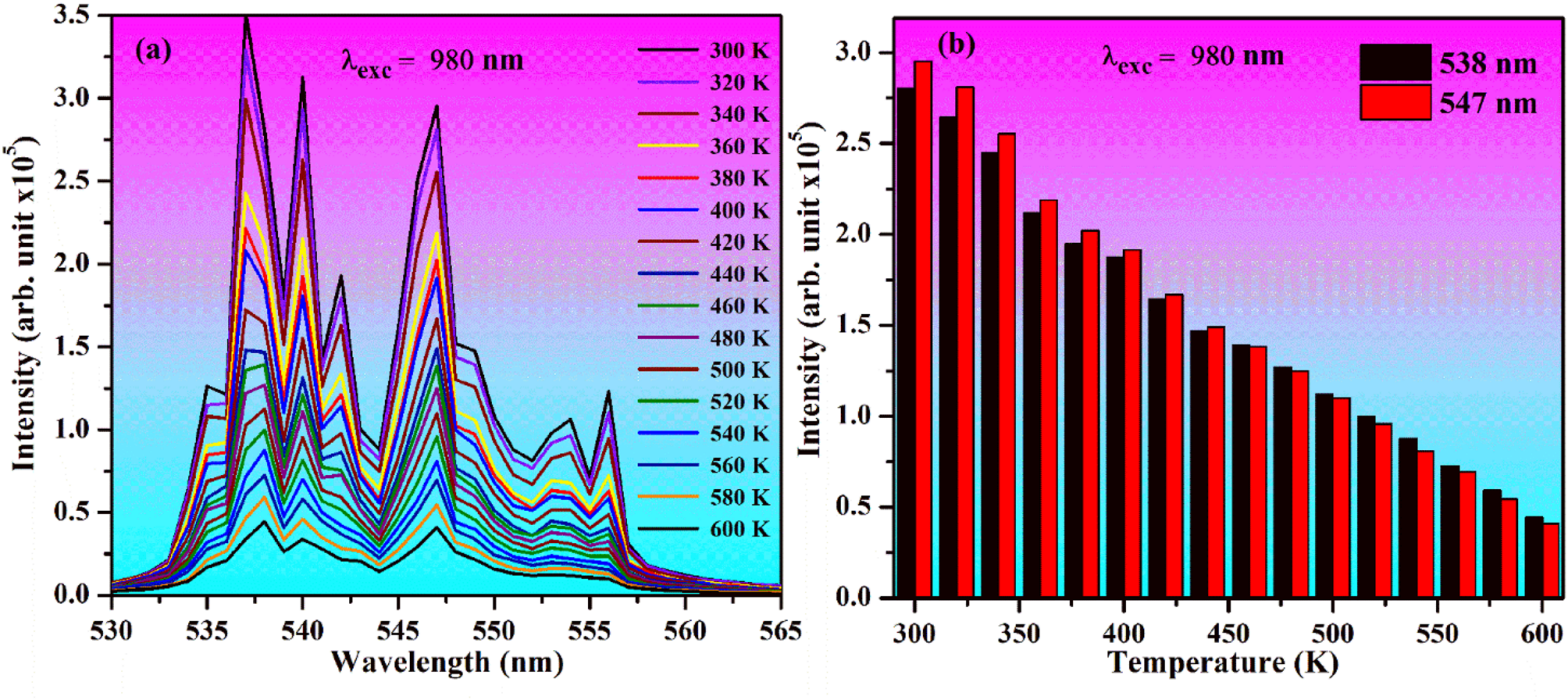
(**a**) Temperature dependent UC spectra of Ho^3+^/Yb^3+^/5Bi^3+^ co-doped ZnGa_2_O_4_ sample under 980 nm excitations at 12.73 W/cm^2^. (**b**) Variation in emission intensity of the two TCLs at 538 and 547 nm monitored at various temperatures.

The intensity of emission bands strongly depends on the temperature of phosphor sample, particularly the emission bands originating from two close lying thermally coupled levels (TCLs). The change in emission intensity can be realized by heating the sample externally. The intensity of emission bands changes on increasing temperature of the source^40^. If the TCLs of a lanthanide ion have a small separation it will be affected by a change in the temperature of sample^3,7,13^. It is well known that the green bands of Ho^3+^ ion arise due to the two TCLs, which are separated by 305 cm^−1^. It can sense a change of population between the two TCLs due to external heat given to the sample^13,43^. In our case, we have recorded the UC emission intensity of Ho^3+^/Yb^3+^/5Bi^3+^ co-doped ZnGa_2_O_4_ sample for two TCLs under 980 nm excitations at 12.73 W/cm^2^ in the range of 300–600 K temperature. Figure 12a depicts the temperature dependent UC emission intensity of two TCLs at 538 and 547 nm wavelengths in the region of 530–565 nm. On increasing the temperature from 300 to 600 K, a population shift of the excited ions takes place from lower level to the upper; however, the peak position of the bands remains unchanged. It is evident that the emission intensity of two TCLs decreases gradually with the rise in temperature^44^.

The emission peak arising from ^5^F_4_ level has two close lying Stark components at 537 and 538 nm wavelengths. We have selected the emission peak at 538 nm to estimate a change in the emission intensity with a temperature because it follows Boltzmann distribution law. In the beginning, the UC intensity of 547 nm is larger while it is smaller for 538 nm. Once the external temperature of phosphor is increased, the UC intensity of 547 nm decreases whereas it is found to increase for 538 nm. At 460 K, the emission intensities of both the peaks are almost the same. On increasing the temperature above 460 K, the intensity of 538 nm emission band is more than 547 nm. However, the overall emission intensities of two TCLs are decreased regularly with temperature. Similarly, Mahata et al^45^ have also used the TCLs of Ho^3+^ ion at 538 and 548 nm wavelengths and found that the UC intensity of two TCLs varies with the rise in temperature.

The UC emission intensities of TCLs at 538 and 547 nm wavelengths at various temperatures are given in Fig. 12b. The figure also clarifies that the emission intensity of 538 nm is initially smaller than 547 nm (during 300–440 K). The UC emission intensities of two peaks are almost equal at 460 K. However, the emission intensity of 538 nm peak is larger than 547 nm peak in the temperature range of 480–600 K. The change in emission intensity between 538 and 547 nm peaks can be taken to calculate the fluorescence intensity ratio (FIR), which is the basis for temperature sensitivity calculation^13,43^.

**Figure 13:**
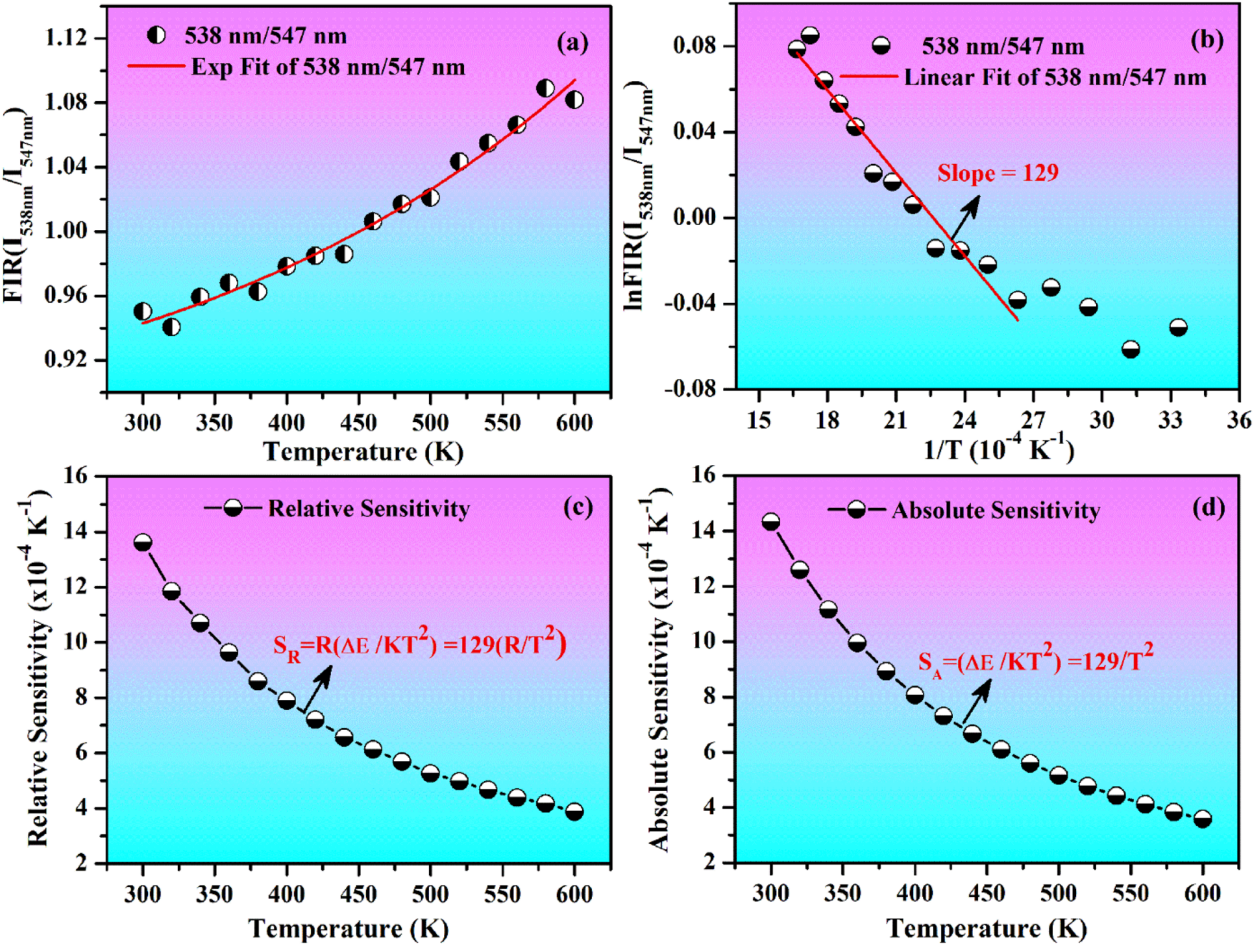
(**a**) Plots of FIR(I_538nm_/I_547nm_) versus temperature, (**b**) lnFIR(I_538nm_/I_547nm_) ~ T^−1^ (**c**) Relative sensitivity ~ temperature and (**d**) Absolute sensitivity ~ temperature for the Ho^3+^/Yb^3+^/5Bi^3+^ co-doped ZnGa_2_O_4_ phosphor under 980 nm excitations at 12.73 W/cm^2^.

Figure 13a shows a plot between FIR (I_538nm_/I_547nm_) of TCLs and the temperature for the Ho^3+^/Yb^3+^/5Bi^3+^ co-doped phosphor under the excitation of 980 nm at 12.73 W/cm^2^. The FIR value rises from 0.95 to 1.08 with increasing temperature from 300 to 600 K, respectively. The nature of FIR (I_538nm_/I_547nm_) slope has been observed exponentially. The FIR values for (^5^F_4_) and (^5^S_2_) levels of Ho^3+^ ion follow Boltzmann distribution law and these values have been evaluated by using the following relation^4,13,40^:

vii $${\text{FIR}} = \frac{{I_{2} }}{{I_{1} }} = {\text{Be}}^{ - \vartriangle E/kT} + C$$

where I_1_ and I_2_ stand for the emission intensity of two peaks arising from lower and upper TCLs, (k = 0.695 cm^−1^ K^−1^) is Boltzmann’s constant, △E is the energy difference between TCLs (i.e. ^5^F_4_ and ^5^S_2_ levels) and T refers to absolute temperature, respectively. Figure 13a also indicates that the values of FIR increase noticeably on increasing temperature of the ZnGa_2_O_4_ phosphor. Figure 13b reveals a plot of ln(FIR) versus (T^−1^) and it also follows the Boltzmann distribution law. This plot gives a slope value of 129 by linear fitting of the experimental data. The obtained value has been taken to calculate the temperature sensitivity in the both cases. Figure 13c,d show the plots between the relative (S_R_) and absolute (S_A_) temperature sensing sensitivities versus temperature for Ho^3+^/Yb^3+^/5Bi^3+^ co-doped sample, respectively. The temperature sensing sensitivities have been evaluated by taking the following relations^13,43,46^:

viii $$S_{R} = \frac{{d\left( {R } \right)}}{d\left( T \right)} = R\left( {\frac{\Delta E}{{kT^{2} }}} \right)$$

ix $$S_{A} = \frac{ 1}{{ R}} \frac{{d\left( {R } \right)}}{d\left( T \right)} = \left( {\frac{\Delta E}{{kT^{2} }}} \right)$$

where the given terms show their usual meanings. The term ‘R’ is fluorescence intensity ratio (FIR) for the two peaks at 538 and 547 nm. We have calculated the relative and absolute sensitivities for different temperatures. The relative sensitivities are found to be 13.6 × 10^−4^ and 3.9 × 10^−4^ K^−1^ while the absolute sensitivities are 14.3 × 10^−4^ and 3.6 × 10^−4^ K^−1^ at 300 and 600 K temperatures, respectively. However, Mahata et al^45^ have reported the temperature sensitivity of 2.0 × 10^−4^ K^−1^ at 300 K in the Ho^3+^/Yb^3+^ activated BaTiO_3_ phosphor. Thus, the temperature sensing sensitivities of the prepared phosphor are better at lower temperature in the present case.

The values of temperature sensing sensitivity have also been observed by many groups of workers in various host materials^17,45,47–55^. We have also carried out a comparison of the temperature sensing sensitivity achieved in our case with the reported values by the other workers in Table 1. It has been concluded from this table that our temperature sensing sensitivity values are very close to the other reported values.

**Table 1:** Comparative analysis of temperature sensitivity values found in the present case and reported by the other workers.

Rare earths and materials	Transition	Temperature (K)	Sensitivity (S) (K^−1^) × 10^−4^ at temp. (K)	References
Ho^3+^–Yb^3+^ : Gd_2_O_3_	^5^F_4_/^5^S_2_ → ^5^I_8_	300–800	18.0 (580) (S_R_) 13.0 (798) (S_A_)	^17^
Ho^3+^–Yb^3+^ : BaTiO_3_	^5^F_4_/^5^S_2_ → ^5^I_8_	12–300	2.0 (300) (S_R_)	^45^
Ho^3+^/Yb^3+^ (Glass–ceramic)	^5^F_2,3_/^3^K_8_,^5^F_1_/^5^G_6_ → ^5^I_8_	303–643 and 1119	10.2 (1119) (S_R_)	^47^
Ho^3+^/Yb^3+^: Sr_3_Y(PO_4_)_3_	^5^I_4_/^5^F_5_ → ^5^I_8_	298–573	4.6 (573) (S_A_)	^48^
Tm^3+^/Yb^3+^: Sr_3_Y(PO_4_)_3_	^3^F_2,3_/^1^G_4_ → ^3^H_6_	298–573	15.3 (573) (S_A_)	^48^
Nd^3+^ (Glass–ceramic)	^4^ F_5/2_/^4^F_3/2_ → ^4^I_9/2_	300–700	15.0 (600) (S_R_)	^49^
Ho^3+^/Tm^3+^/Yb^3+^: Ba_3_Y_4_O_9_	^5^F_5_/^5^F_4_,^5^S_2_ → ^5^I_8_	294–573	17.0 (573) (S_A_)	^50^
Yb^3+^–Er^3+^–Mg^2+^: Ca_3_Al_2_O_6_	^2^H_11/2_/^4^S_3/2_ → ^4^I_15/2_	0–2000	78.0 (145) (S_R_)	^51^
Mn^4+^: Na_2_WO_2_F_4_	^2^E_g_ → ^4^A_2g_	0–800	65.0 (193) (S_R_)	^52^
Tm^3+^/Yb^3+^: YF_3_	^3^F_2_,_3_ → ^3^H_6_	300–750	10.1 (750) (S_A_)	^53^
Er^3+^/Yb^3+^: Y_2_O_3_	^2^H_11/2_/^4^S_3/2_ → ^4^I_15/2_	93–613	44.0 (427) (S_R_)	^54^
Er^3+^/Yb^3+^: Al_2_O_3_	^2^H_11/2_/^4^S_3/2_ → ^4^I_15/2_	295–973	51.0 (495) (S_R_)	^55^
Ho^3+^/Yb^3+^/ Bi^3+^: ZnGa_2_O_4_	^5^F_4_/^5^S_2_ → ^5^I_8_	300–600	13.6 (300) (S_R_) 3.9 (600)	Present work
Ho^3+^/Yb^3+^/Bi^3+^: ZnGa_2_O_4_	^5^F_4_/^5^S_2_ → ^5^I_8_	300–600	14.3 (300) (S_A_) 3.6 (600)	Present work

### Color tunability, CCT and color purity analyses

**Figure 14:**
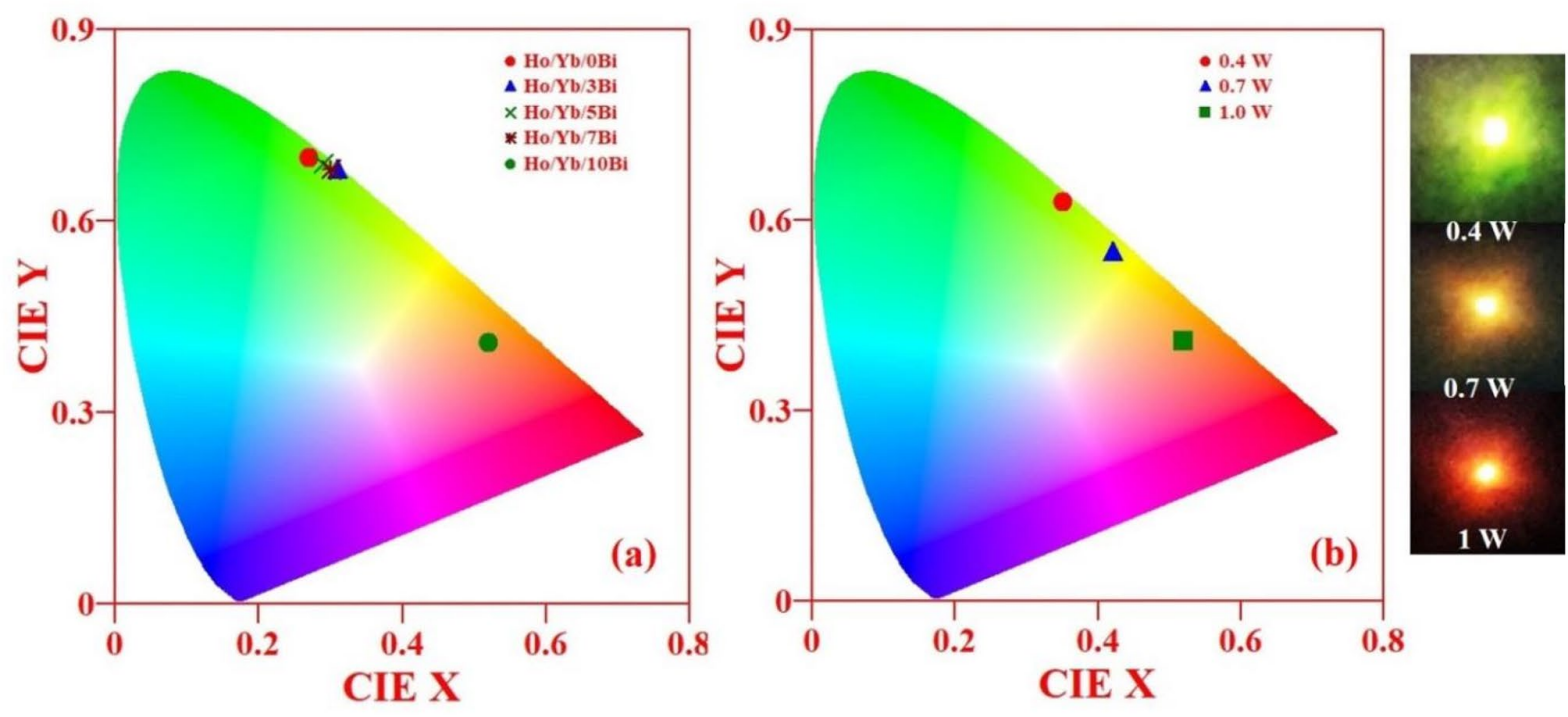
The CIE diagrams for (**a**) the Ho^3+^/Yb^3+^/xBi^3+^ co-doped ZnGa_2_O_4_ phosphor materials for various concentrations of Bi^3+^ ion (i.e. x = 0, 3, 5, 7 and 10 mol%) under 980 nm excitations at 31.84 W/cm^2^ and (**b**) for various power densities of 980 nm diode laser (i.e. 12.73, 22.29 and 31.84 W/cm^2^) in the Ho^3+^/Yb^3+^/10Bi^3+^ co-doped material using GoCIE 1931 software.

The Commission International de l’Eclairage coordinates (CIE) have x and y parameters to determine the color tunability. The CIE diagram shows the hue and saturation in the two dimensional coordinates, which is also termed as the chromaticity diagram. The CIE diagrams were plotted for various concentrations of Bi^3+^ ion (i.e. x = 0, 3, 5, 7 and 10 mol%) in the Ho^3+^/Yb^3+^ co-doped materials under 980 nm excitations at 31.84 W/cm^2^ and also for the 12.73, 22.29 and 31.84 W/cm^2^ power densities of 980 nm in the Ho^3+^/Yb^3+^/10Bi^3+^ co-doped sample with the help of GoCIE 1931 software. Figure 14a reveals the CIE diagram of the Ho^3+^/Yb^3+^/xBi^3+^ (i.e. x = 0, 3, 5, 7 and 10 mol%) co-doped phosphor materials under 980 nm excitations at 31.84 W/cm^2^. The color emitted by phosphor materials is found to tune from green to red via yellow regions^4^. The CIE coordinates change from (0.27, 0.70) to (0.52, 0.41) on varying Bi^3+^ ion concentrations. This confirms that the color tunability in Ho^3+^/Yb^3+^ co-doped phosphor materials has been obtained through Bi^3+^ doping.

On the other hand, the emitted color of phosphor can also be changed on varying the power density of 980 nm source^25^. The CIE diagram of Ho^3+^/Yb^3+^/10Bi^3+^ co-doped material at various power densities of 980 nm i.e. 12.73, 22.29 and 31.84 W/cm^2^ is shown in Fig. 14b. At lower power density i.e. 12.73 W/cm^2^, the color of phosphor is green, which becomes yellow at 22.29 W/cm^2^. On further increase in the power density from 22.29 to 31.84 W/cm^2^, the emitted color is tuned from the yellow to red regions^4,56^. Therefore, the color coordinates of phosphors vary considerably with the rise in concentration and power density. The calculated CIE coordinates thus obtained in the two cases are also summarized in Table 2.

**Table 2:** Variations of CIE coordinates, CCT values and color purity with various concentrations of Bi^3+^ ion in the Ho^3+^/Yb^3+^ co-doped materials and at various power densities of 980 nm excitations for the Ho^3+^/Yb^3+^/10Bi^3+^ co-doped material.

Concentration of Bi^+^ ions (mol%)	CIE coordinates (x, y)	CCT (K)	Color purity (%)	Power density (W/cm^2^) for Ho/Yb/10Bi phosphor	CIE coordinates (x, y)	CCT (K)	Color purity (%)
Ho/Yb/0Bi	(0.27, 0.70)	6395	93.8	12.73	(0.35, 0.63)	5249	94.9
Ho/Yb/3Bi	(0.31, 0.68)	5831	98.6				
Ho/Yb/5Bi	(0.29, 0.69)	6113	96.1	22.29	(0.42, 0.55)	4071	91.4
Ho/Yb/7Bi	(0.30, 0.68)	5976	95.8				
Ho/Yb/10Bi	(0.52, 0.41)	2012	79.1	31.84	(0.52, 0.41)	2012	79.1

Basically, the CCT refers to correlated color temperature and it is used to show cool and warm nature of light. By using CIE coordinates (x, y) of the phosphors, we have also evaluated the CCT with the help of McCAMY’s formula. The CCT equation is written below^4^:

x $${\text{CCT}} = 449\;{\text{n}}^{3} + 3525\;{\text{n}}^{2} + 6823.3\;{\text{n}} + 5520.33$$

where n = (x − 0.3320)/(0.1858 − y) and (x, y) refer to the calculated values of CIE coordinates for the phosphors. The CCT values are obtained as 6395, 5831, 6113, 5976 and 2012 K for the Ho^3+^/Yb^3+^/xBi^3+^ (i.e. x = 0, 3, 5, 7 and 10 mol%) co-doped phosphor materials, respectively. The CCT value varies from cool light to the extra warm light. On varying the power density, the values of CCT of the Ho^3+^/Yb^3+^/10Bi^3+^ co-doped ZnGa_2_O_4_ material are found to be 5249, 4071 and 2012 K for the 12.73, 22.29 and 31.84 W/cm^2^, respectively (see Table 2). It shows that the CCT value also shifts from the natural light to the extra warm light with the power density^57,58^. Therefore, the Ho^3+^/Yb^3+^/Bi^3+^ co-doped ZnGa_2_O_4_ phosphor is stable material and may be used for the applications of cool and warm LEDs.

Color purity is also one of the important parameter to realize the performance of a phosphor. The color purity has been calculated by the following relation^59–61^:

xi $${\text{Color}}\;{\text{purity}} = \frac{{\sqrt {(x - x_{i} )^{2 } + (y - y_{i} )^{2 } } }}{{\sqrt {(x_{d} - x_{i} )^{2 } + (y_{d} - y_{i} )^{2 } } }} \times 100\%$$

where (x, y), (x_i,_ y_i_) and (x_d_, y_d_) are the CIE coordinates of phosphor, the standard light source and dominant wavelength, respectively. The values of color purity of the Ho^3+^/Yb^3+^/xBi^3+^ (i.e. x = 0, 3, 5, 7 and 10 mol%) co-doped phosphors are calculated to be 93.8, 98.6, 96.1, 95.8 and 79.1%, respectively (see Table 2). The color purity of phosphor is smaller for the Ho^3+^/Yb^3+^ doped sample. However, it is observed to increase through doping of Bi^3+^ ion. At higher concentrations of Bi^3+^ ion, the color purity is decreased^61^.

On the other hand, the color purity of Ho^3+^/Yb^3+^/10Bi^3+^ co-doped sample also decreases on varying the power density of 980 nm diode laser from 12.73 to 31.84 W/cm^2^^59^. At low power density i.e. 12.73 W/cm^2^, the color purity of phosphor is found to be 94.9%. When the power density is changed from 22.29 to 31.84 W/cm^2^, the obtained values of color purity is decreased from 91.4 to 79.1%, respectively (see Table 2). It is clear from the above that color purity of a phosphor material is dependent on the Bi^3+^ ion concentrations and the power density. The lower value of the color purity refers to a shifting of the emitted light towards white region of the CIE diagram. Thus, the high color purity has been achieved to 98.6% in the phosphor through Bi^3+^ doping.

## Conclusions

The Ho^3+^/Yb^3+^/xBi^3+^ co-doped ZnGa_2_O_4_ phosphor materials have been prepared by using solid state reaction method. The XRD analyses give an idea about the phase and crystalline nature of phosphors. The UV–vis–NIR absorption spectra show different bands of the Ho^3+^, Yb^3+^ and Bi^3+^ ions in the phosphors. The band gap of Ho^3+^/Yb^3+^ co-doped phosphor is reduced via doping of Bi^3+^ ion. The Ho^3+^ doped ZnGa_2_O_4_ phosphor emits intense green color under 980 nm excitations. The emission intensity of green band of the Ho^3+^ doped phosphor is increased upto 128 and 228 times through co-doping of Yb^3+^ and Yb^3+^/Bi^3+^ ions, respectively. This is attributed to energy transfer and improvement in local crystal structure of the phosphor. The relative and absolute temperature sensing sensitivities of Ho^3+^/Yb^3+^/5Bi^3+^ co-doped phosphor are found as 13.6 × 10^−4^ and 14.3 × 10^−4^ K^−1^ at 300 K, respectively. The CIE diagrams of phosphors show excellent color tunability with high color purity of 98.6% through doping of Bi^3+^ ion. The CCT value of phosphors shifts from the cool light to the extra warm light. Therefore, the Ho^3+^/Yb^3+^/Bi^3+^ co-doped ZnGa_2_O_4_ phosphors can be useful in UC based color tunable devices, green LEDs and as temperature sensors.

## Experimental method

### Synthesis

The Ho^3+^, Yb^3+^ and Bi^3+^ doped and co-doped ZnGa_2_O_4_ phosphors have been prepared by solid state reaction method^4^. The starting materials used were Ho_2_O_3_ (99.99%), Yb_2_O_3_ (99.99%), ZnO (99.99%), Ga_2_O_3_ (99.99%) and Bi_2_O_3_ (99%). The Ho^3+^/Yb^3+^/xBi^3+^ co-doped phosphors have been prepared with the fixed concentrations of Ho^3+^ and Yb^3+^ ions and these are kept at 1 and 3 mol%, respectively. The concentrations of Bi^3+^ ion were varied as x = 3, 5, 7 and 10 mol%. The starting materials were weighed carefully and mixed completely in the agate mortar by taking acetone as a mixing agent. The homogeneously mixed powder was placed in an alumina crucible and then heated within the closed furnace at 1200 °C for 5 h. The heating temperature was constant for all the materials. The obtained materials are crushed properly in the agate mortar to form fine powders. The obtained powders are used for the structural and optical studies.

### Instrumentation

The XRD measurements were carried out to study the crystalline nature and phase purity of the phosphor materials by using CuKα radiation (λ = 0.15406 nm) based Rigaku diffractometer system. The surface morphology of phosphor was studied by SEM (Zeiss, Evo18 Research). The presence of different constituents in the phosphor samples was documented by EDS technique. The EDS mapping images were generated by using INCA software attached with INCAx-act Oxford Instruments (51-ADD0048). The UV–vis–NIR absorption spectra were studied in diffuse reflectance mode with the help of Perkin Elmer Lambda-750 (Ultraviolet–visible-Near infrared spectrometer) unit in the 200–1100 nm region. The FTIR spectra were monitored by using a Perkin Elmer IR spectrometer (I Frontier unit) in 400–4000 cm^−1^ range. The upconversion emission spectra were monitored with the help of 980 nm and also iHR320 Horiba Jobin Yvon spectrometer attached with PMT. The decay curves for ^5^F_4_ level of the Ho^3+^ ion were monitored by chopping continuous beam of 980 nm radiations with the help of a mechanical chopper and 150 MHz digital oscilloscope of Hameg instruments using Model No. HM1507. Finally, the phosphor materials were heated outside with the digital thermo-couple arrangements for analyzing the temperature sensing capability. The CIE diagrams of the phosphor samples were drawn with the help of GoCIE 1931 software.
